# Micro-RNA Analysis of Pancreatic Cyst Fluid for Diagnosing Malignant Transformation of Intraductal Papillary Mucinous Neoplasm by Comparing Intraductal Papillary Mucinous Adenoma and Carcinoma

**DOI:** 10.3390/jcm10112249

**Published:** 2021-05-22

**Authors:** Yohei Shirakami, Takuji Iwashita, Shinya Uemura, Hisashi Imai, Katsutoshi Murase, Masahito Shimizu

**Affiliations:** 1 First Department of Internal Medicine, Gifu University Hospital, 1-1 Yanagido, Gifu 501-1194, Japan; takuji@w7.dion.ne.jp (T.I.); ueshin550621@gmail.com (S.U.); shimim-gif@umin.ac.jp (M.S.); 2 Department of Digestive Surgery, Gifu University Hospital, 1-1 Yanagido, Gifu 501-1194, Japan; h_imai@gifu-u.ac.jp (H.I.); k_murase@gifu-u.ac.jp (K.M.)

**Keywords:** IPMN, micro-RNA, pancreatic cyst fluid, EUS-FNA, pancreatic cancer

## Abstract

Although intraductal papillary mucinous neoplasm (IPMN) is thought to be a precursor lesion of pancreatic cancer, diagnosing malignant transformation of IPMN using non-invasive diagnostic methods is difficult and complicated. Micro-RNAs (miRNAs) are currently recognized as biomarkers and molecular targets of various diseases, including malignancy. In this study, we investigated a potential diagnostic approach using miRNA in pancreatic cyst fluid as a marker for evaluating malignant alternation of IPMN. Cystic fluid was sampled mainly during surgical resection. The collected samples were evaluated by performing comprehensive analysis of miRNA using a highly sensitive DNA chip. miRNA expression was compared between IPM adenoma (IPMA) and IPM carcinoma (IPMC) to evaluate the related biomarkers for malignant transformation of IPMN. miRNA analysis revealed that six miRNAs (miR-711, miR-3679-5p, miR-6126, miR-6780b-5p, miR-6798-5p, and miR-6879-5p) in IPMC were significantly enriched compared to those in IPMA. The difference was validated using quantitative real-time PCR. Cyst fluid miRNA analysis might be useful for diagnosing malignant alteration of IPMN. Further evaluations of diagnostic capability as well as functional analysis using the identified miRNAs are required with larger cohorts to confirm its efficacy.

## 1. Introduction

In recent years, incidental detection of cystic lesions in the pancreas has increased due to the development of medical equipment and frequent imaging examination [1]. Among pancreatic cysts, intraductal papillary mucinous neoplasm (IPMN) is considered a precursor lesion of pancreatic cancer; therefore, accurate differential diagnosis through careful follow-up using imaging studies of IPMN is considered important to detect early-stage pancreatic cancer. It is, however, not straightforward malignant alternation of IPMN even with standard axial imaging studies, such as computed tomography or magnetic resonance imaging, or cyst fluid analysis including the carcinoembryonic antigen (CEA) level or cytology. Therefore, development of appropriate diagnostic biomarkers is needed for the management of IPMNs [2].

Micro-RNA (miRNA), a type of functional RNA, is mainly present in exosomes and is considered to be involved in regulating gene expression, which in turn contributes to cellular signal transduction [3,4]. It has been widely examined whether miRNAs can be potential biomarkers for the detection, diagnosis, and prognosis of various diseases [5,6]. This study area has shown rapid evolution, especially in the field of cancer research [7,8,9]. miRNAs are known to be stable nucleic acids that are measurable in tissues and body fluids, including plasma, urine, saliva, tears, peritoneal fluid, pleural fluid, and cerebrospinal fluid [10]. In addition, the miRNA signature appears to be specific to various types of cells; therefore, featured miRNAs secreted by each cell can reach the peripheral tissues and can be detected on examining tissues and fluid. Sampling of these miRNAs in blood and measuring, which is a type of liquid biopsy, is considered useful in reducing the necessity of invasive manipulation [10].

Here, we present a pilot study with a comprehensive analysis of miRNAs in pancreatic cyst fluid. The aim of this study was to investigate the ability of the potential diagnostic approach by using pancreatic cyst fluid miRNA as a marker for evaluating the malignant transformation of IPMN.

## 2. Materials and Methods

### 2.1. Collection of Pancreatic Cyst Fluid

Pancreatic cyst fluid specimens were sampled from patients during endoscopic ultrasound-guided fine needle aspiration (EUS-FNA), endoscopic retrograde cholangio-pancreatography (ERCP), or surgical resection, all of which were performed between January 2016 and July 2017 at Gifu University Hospital and led to incidental diagnosis of a pancreatic cyst. The collected cyst fluid was stored at −80 °C until analysis. IPMN was defined as a multi-lobular mucinous cystic lesion communicating with the pancreatic duct. The diagnosis of malignant alternation was determined based on the pathological diagnosis of the surgically resected specimen. If the pathological diagnosis was not available, especially in negative cases for cancer, the final diagnosis was made based on clinical information with at least six months’ follow-up. All patients provided written informed consent for the study. This study was carried out in accordance with the Declaration of Helsinki. The study protocol was approved by the institutional review board of Gifu University.

### 2.2. RNA Extraction and miRNA Expression Profiling

RNA was extracted from pancreatic cyst fluid using the 3D-Gene^TM^ RNA extraction reagent from liquid samples (Toray Industries, Tokyo, Japan), according to the manufacturer’s instructions. Extracted total RNA was checked using a Bioanalyzer (Agilent, Santa Clara, CA, USA) and labeled with the 3D-Gene^TM^ miRNA labeling kit (Toray Industries). Half volumes of labeled RNAs were hybridized onto the 3D-Gene^TM^ Human miRNA Oligo chip (Toray Industries). The annotation and oligonucleotide sequences of the probes conformed to the miRbase miRNA database (http://www.mirbase.org/, accessed on 5 February 2018). After stringent washes, fluorescent signals were scanned with the 3D-Gene^TM^ Scanner (Toray Industries) and analyzed using 3D-Gene^TM^ Extraction software (Toray Industries). The raw data of each spot was normalized by substitution with a mean intensity of the background signal determined by all blank spots’ signal intensities at 95% confidence intervals. Measurements of spots with signal intensities greater than two standard deviations of the background signal intensity were considered valid. The relative expression level of a given miRNA was calculated by comparing the signal intensities of the valid spots throughout the microarray experiments. The normalized data were globally normalized per array, such that the median of the signal intensity was adjusted to 25.

### 2.3. Real Time RT-PCR for Quantifying miRNA

Total miRNA was extracted from pancreatic cyst fluid samples using the Qiagen miRNeasy kit (Qiagen, Venlo, the Netherlands), according to the manufacturer’s instructions. The TaqMan^TM^ MicroRNA Reverse Transcription Kit (Applied Biosystems, Waltham, MA, USA) was used to convert the selected miRNAs into cDNA using a LightCycler (Roche Diagnostics, Indianapolis, IN, USA). As controls for the extraction and amplification steps, *Caenorhabditis elegans* miR-39 (cel-miR-39) was spiked into each sample. The reverse transcription product was amplified using primers and probes provided by Applied Biosystems using TaqMan^TM^ MicroRNA Assay, according to the manufacturer’s instructions. The expression levels of miRNA were measured in triplicate.

### 2.4. Statistical Analyses

Statistical analyses were conducted using R ver. 3.3.1 (R Foundation for Statistical Computing; http://www.R-project.org/, accessed on 14 February 2018). The miRNA data were analyzed using the Mann–Whitney U test with a false discovery rate <10% after quantile normalization. A paired analysis between the groups was performed using the Mann–Whitney U test. Data are expressed as box-and-whisker plots with median values. Boxes extend from the 25th to the 75th percentile of each group’s distribution of values. Vertical extending lines mean adjacent values, i.e., the most extreme values within a 1.5 interquartile range of the 25th and 75th percentile of each group, and values beyond these upper and lower bounds are considered outliers. A *p*-value < 0.05 indicated a significant difference between the groups.

## 3. Results

### 3.1. Descriptive

**Figure 1:**
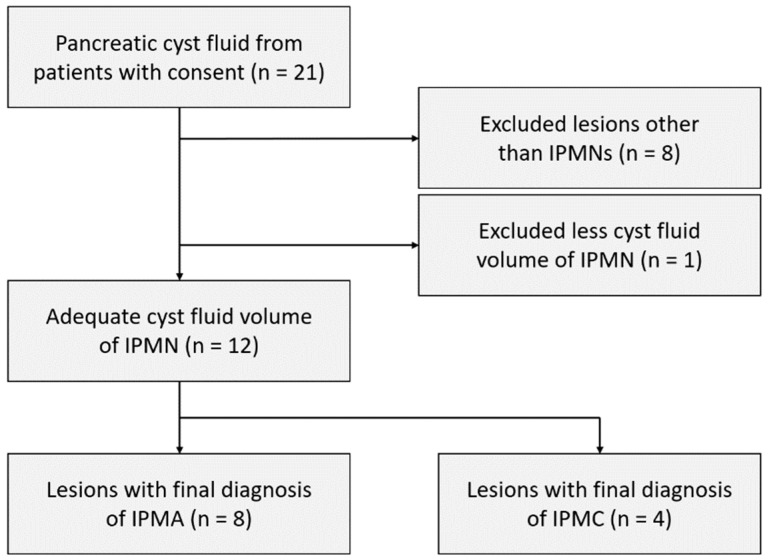
Flow chart of the study samples. IPMA, intraductal papillary mucinous adenoma; IPMC, intraductal papillary mucinous carcinoma; IPMN, intraductal papillary mucinous neoplasm.

A total of 21 pancreatic cyst fluid samples were sampled during the study period. Of these, 13 lesions were diagnosed as IPMNs, including intraductal papillary mucinous adenoma (IPMA) and intraductal papillary mucinous carcinoma (IPMC), by histological validation of surgical specimens or the definition of IPMN, described below. A minimum of 600 μL fluid was needed for comprehensive analysis of miRNA; therefore, one sample with a volume less than 600 μL was excluded. The remaining 12 specimens were included in the present study to investigate the possibility of miRNAs in cyst fluid as diagnostic markers to predict malignant transformation of IPMN (Figure 1).

Cystic lesions of IPMNs were located in the head of the pancreas in two patients, the neck in one, the body in six, tail in two, and in the main pancreatic duct in one. The median longest diameter of the cysts was 39 mm. Cystic fluid was obtained during EUS-FNA in one patient, ERCP in four, and surgery in seven. The final diagnoses were IPMA in eight patients and IPMC in four. The clinical and pathological features of IPMN lesions are summarized in Table 1. More details regarding the cyst characteristics and comparing between IPMA and IPMC are shown in Table S1.

### 3.2. miRNA Expression Profiling and Comparing between IPMA and IPMC

**Figure 2:**
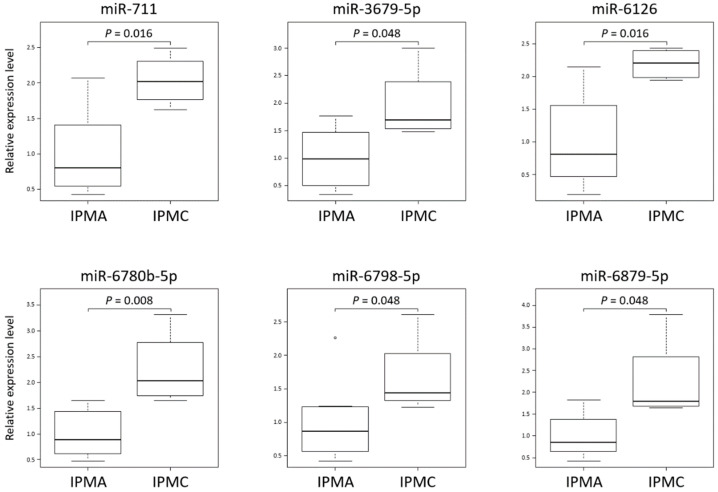
Comprehensive analysis of miRNA expression in cyst fluid of IPMN and comparing IPMA with IPMC. RNA was extracted from IPMN cyst fluid and labeled. Then, RNAs were hybridized and fluorescent signals were scanned as described in Materials and Methods. A relative expression level of a given miRNA was calculated by comparing the signal intensities of the valid spots. The data were normalized by the expression levels of miRNA in IPMA cyst fluid. Among the targeting 2565 genes, indicated six in cyst fluid from IPMC were significantly higher level than those in IPMA. The data were analyzed by Mann–Whitney U test. Data are expressed as box-and-whisker plots. Within the boxes, the center lines denote the median values and boxes extend from the 25th to the 75th percentile of each group’s distribution of values. Vertical extending lines mean adjacent values, i.e., the most extreme values within 1.5 interquartile range of the 25th and 75th percentile of each group, and the value beyond the upper and lower bounds is considered an outlier marked with a white dot. A *p*-value < 0.05 considered a significant difference between the groups. IPMA, intraductal papillary mucinous adenoma; IPMC, intraductal papillary mucinous carcinoma.

Comprehensive miRNA analysis of IPMN cyst fluid was performed targeting 2565 genes; among them, 656 genes could be analyzed and compared. Of these, six miRNAs in cyst fluid from IPMC were significantly higher than those in IPMA, which included miR-711, miR-3679-5p, miR-6126, miR-6780b-5p, miR-6798-5p, and miR-6879-5p (Figure 2).

### 3.3. Quantifying the Expression Levels of miRNAs Using Real-Time RT-PCR

**Figure 3:**
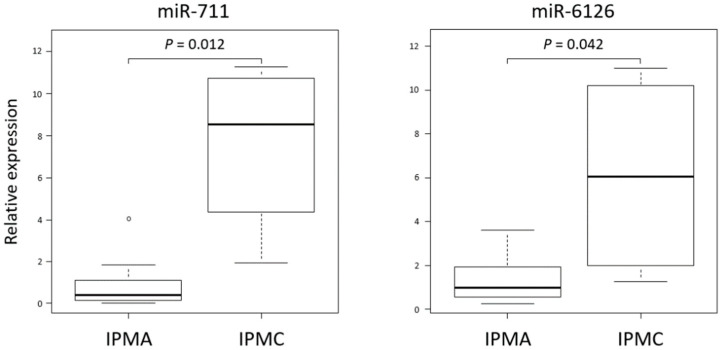
Quantifying the expression levels of miRNAs by real-time RT-PCR and validating the difference between IPMA and IPMC. Indicated two miRNAs in cyst fluid from IPMC were significantly higher level than those in IPMA. The data were analyzed by Mann–Whitney U test. Data are expressed as box-and-whisker plots. Within the boxes, the center lines denote the median values and boxes extend from the 25th to the 75th percentile of each group’s distribution of values. Vertical extending lines mean adjacent values, i.e., the most extreme values within 1.5 interquartile range of the 25th and 75th percentile of each group, and the value beyond the upper and lower bounds is considered an outlier marked with a white dot. A *p*-value < 0.05 considered a significant difference between the groups. IPMC, intraductal papillary mucinous carcinoma; IPMN, intraductal papillary mucinous neoplasm.

To validate the results of the comprehensive miRNA analysis and to quantify the expression levels of miRNA, real-time RT-PCR was performed. In the present study, several miRNAs described above, including miR-711 and miR-6126, were quantified by real-time RT-PCR and compared between IPMC and IPMA. Consistent with the comprehensive analysis, the expression levels of these miRNAs were found to be significantly upregulated in the specimens from IPMC cyst fluid than in those from IPMA (Figure 3).

## 4. Discussion

Although IPMN is considered a precursor lesion of pancreatic cancer, the gold standard method to accurately diagnose malignant transformation of IPMN without surgical resection is unknown. Various diagnostic methods, which are considered relatively less invasive, have been tested to distinguish pre-malignant (IPMA) or malignant (IPMC) lesions. These include cytology using EUS-FNA and the value of CEA and DNA mutation analyses from cyst fluid; however, the diagnostic performance has been found to be inadequate [11,12,13,14]. Therefore, development of appropriate diagnostic biomarkers is needed for the management of IPMNs [2]. The present study demonstrated a potential diagnostic approach by using miRNA in pancreatic cyst fluid as a marker for evaluating malignant transformation of IPMN.

In this study, we performed a comprehensive analysis of miRNA expression patterns in pancreatic cyst fluid obtained from IPMNs. The analysis revealed six miRNAs that were differentially enriched in fluid samples from patients with IPMC in comparison to those with IPMA. Previous studies have demonstrated a number of deregulated miRNAs in tissue and circulating blood of patients with PDAC as well as IPMN [15,16,17,18]. In addition, differential abundance of a number of miRNAs among various types of pancreatic cyst fluid, including IPMN [19,20,21]. The miRNAs (miR-711, miR-3679-5p, miR-6126, miR-6780b-5p, miR-6798-5p, and miR-6879-5p) detected in the present study when comparing IPMC and IPMA were considered quite novel. In other words, these did not appear to be consistent with previously reported ones in the tissue, blood, or cystic fluid, although we hypothesized that altered expression of miRNA in the cyst fluid of IPMC could be similar to that of PDAC. The reasons for this inconsistency might be as follows: first, the pancreatic cyst is a closed cavity and the content of fluid in that area can be different from circulating blood. miRNAs from pancreatic neoplasms, either cancer or adenoma, may be secreted into the blood stream rather than into the cystic cavity. Second, this study, for the first time, specifically focused on comparing IPMA and IPMC. A previous paper mentioned the difference of miRNA between “low grade-benign” and “high grade-invasive” lesions [21], but the method for grouping was not similar to ours. Another report has indicated altered miRNA expression profiles in various types of pancreatic cysts [20]. Although the study revealed many up- or down-regulated miRNAs in IPMN, a comparison was made between IPMN and serous cystadenoma. In addition, the samples used in the study were not cystic fluid, but formalin-fixed and paraffin-embedded tissues.

In the field of cancer research, it is thought that some miRNAs function as oncogenic miRNAs (i.e., oncomiR) and others as tumor suppressors by targeting various genes [3,4]. For example, miR-21 and miR-23a were identified as oncomiRs in several types of cancer, which inhibit the functions of tumor suppressor genes, and these miRNAs can be diagnostic and prognostic biomarkers [22,23]. As a tumor suppressor, miR-204 has been reported to inhibit migration and invasion in several cancer cell lines [24,25]. A previous report has also demonstrated that a tumor suppressor miR-34a was decreased in various types of cancer [26]. As described above, miRNAs revealed in the present study are uncommon and have never been reported, at least in the area of cancer study; therefore, we tried to clarify their functions through the miRDB MicroRNA Target Prediction and Functional Study Database (http://www.mirdb.org/, accessed on 3 April 2019). Although several target genes of each miRNA were detected, genes associated with cancer promotion or suppression could not be identified. Further research should be conducted to reveal the role and function of these miRNAs in the development of pancreatic cancer.

Despite the novelty of this study, there are limitations to be considered. This study was conducted at a single center and included a small number of patients. Due to an inadequate sample size, more detailed analyses, including a multivariate analysis with cyst characteristics and clinical data, were not possible. The present study also could not evaluate the association between miRNA and cancerous transformation; therefore, this pilot trial is considered as a proof of concept using cyst fluid miRNAs rather than as a proof of the association above. More sample size might enable us to predict the presence of identified miRNAs in the lesions and to estimate the relationship between cancerous types and miRNAs. In addition, a prospective cohort study will be able to validate the usefulness of those miRNAs as biomarkers, which reveal a critical long-term merit of this study. Moreover, histopathological examination for accurate diagnosis was not performed in all patients. With respect to the validation study, quantitative PCR was not carried out with all the miRNAs differentiated between IPMC and IPMA. Furthermore, the stability of miRNA has never been examined on exposure to pancreatic juice or bile. Although several papers have been published to indicate the usefulness of miRNA in pancreatic cyst fluid for diagnosis, the stability of miRNA has not been discussed. The mixing proportion of pancreatic juice may affect the profile of miRNA in cyst fluid. Further evaluations of diagnostic capability as well as functional analysis using the identified miRNAs are required with a larger cohort to confirm its efficacy.

## 5. Conclusions

As cystic lesions in the pancreas are being detected more frequently, there is an urgent need for useful biomarkers to manage pancreatic cysts, especially IPMNs. We present a pilot study with comprehensive analysis of miRNAs in pancreatic cyst fluid, demonstrating that IPMC and IPMA have differential miRNA profiles, in which the expression of six miRNAs is markedly elevated in IPMC. These miRNAs appear to be appropriate biomarker candidates to detect malignant transformations in IPMN. In addition, it is possible that sampling cyst fluid using EUS-FNA can be an effective means for managing pancreatic cystic lesions in the future.

## Figures and Tables

**Table 1:** Clinical and pathological features of IPMN lesions.

Total number of patients, n	12
Age, years, median (range)	73 (48–76)
Gender, male/female, n	7/5
Diameter of the lesions mm, median (range)	39 (18–75)
Site of the lesions, head/neck/body/tail/MPD, n	2/1/6/2/1
Collection method, EUS-FNA/ERCP/surgery, n	1/4/7
Diagnosis, IPMA/IPMC, n	8/4

EUS-FNA, endoscopic ultrasound-guided fine needle aspiration; ERCP, endoscopic retrograde cholangiopancreatography; IPMA, intraductal papillary mucinous adenoma; IPMC, intraductal papillary mucinous carcinoma; IPMN, intraductal papillary mucinous neoplasm; MPD, main pancreatic duct.

## Data Availability

Data available on request due to restrictions, e.g., privacy or ethical. The data presented in this study are available on request from the corresponding author.

## Supplementary Materials

The following are available online at https://www.mdpi.com/article/10.3390/jcm10112249/s1, Table S1: Clinical and pathological features of IPMN lesions comparing IPMA with IPMC.

## References

[1] Lee K.S., Sekhar A., Rofsky N.M., Pedrosa I. (2010). Prevalence of incidental pancreatic cysts in the adult population on MR imaging. Am. J. Gastroenterol.

[2] Iwashita T., Uemura S., Mita N., Iwasa Y., Ichikawa H., Senju A., Yasuda I., Shimizu M. (2020). Utility of endoscopic ultrasound and endoscopic ultrasound-guided fine-needle aspiration for the diagnosis and management of pancreatic cystic lesions: Differences between the guidelines. Dig. Endosc..

[3] Croce C.M., Calin G.A. (2005). miRNAs, cancer, and stem cell division. Cell.

[4] Gregory R.I., Shiekhattar R. (2005). MicroRNA biogenesis and cancer. Cancer Res..

[5] Bartel D.P. (2004). MicroRNAs: Genomics, biogenesis, mechanism, and function. Cell.

[6] Duarte F.V., Palmeira C.M., Rolo A.P. (2015). The Emerging Role of MitomiRs in the Pathophysiology of Human Disease. Adv. Exp. Med. Biol..

[7] Bartels C.L., Tsongalis G.J. (2009). MicroRNAs: Novel biomarkers for human cancer. Clin. Chem..

[8] Tutar Y. (2014). miRNA and cancer; computational and experimental approaches. Curr. Pharm. Biotechnol..

[9] Hawa Z., Haque I., Ghosh A., Banerjee S., Harris L., Banerjee S.K. (2016). The miRacle in Pancreatic Cancer by miRNAs: Tiny Angels or Devils in Disease Progression. Int. J. Mol. Sci..

[10] Siravegna G., Marsoni S., Siena S., Bardelli A. (2017). Integrating liquid biopsies into the management of cancer. Nat. Rev. Clin. Oncol..

[11] Maker A.V., Lee L.S., Raut C.P., Clancy T.E., Swanson R.S. (2008). Cytology from pancreatic cysts has marginal utility in surgical decision-making. Ann. Surg. Oncol..

[12] Sawhney M.S., Devarajan S., O’Farrel P., Cury M.S., Kundu R., Vollmer C.M., Brown A., Chuttani R., Pleskow D.K. (2009). Comparison of carcinoembryonic antigen and molecular analysis in pancreatic cyst fluid. Gastrointest. Endosc..

[13] Snozek C.L.H., Mascarenhas R.C., O’Kane D.J. (2009). Use of cyst fluid CEA, CA19-9, and amylase for evaluation of pancreatic lesions. Clin. Biochem..

[14] Khalid A., Zahid M., Finkelstein S.D., LeBlanc J.K., Kaushik N., Ahmad N., Brugge W.R., Edmundowicz S.A., Hawes R.H., McGrath K.M. (2009). Pancreatic cyst fluid DNA analysis in evaluating pancreatic cysts: A report of the PANDA study. Gastrointest. Endosc..

[15] Vila-Navarro E., Vila-Casadesús M., Moreira L., Duran-Sanchon S., Sinha R., Ginés À., Fernández-Esparrach G., Miquel R., Cuatrecasas M., Castells A. (2017). MicroRNAs for Detection of Pancreatic Neoplasia: Biomarker Discovery by Next-generation Sequencing and Validation in 2 Independent Cohorts. Ann. Surg..

[16] Permuth-Wey J., Chen D.-T., Fulp W.J., Yoder S.J., Zhang Y., Georgeades C., Husain K., Centeno B.A., Magliocco A.M., Coppola D. (2015). Plasma MicroRNAs as Novel Biomarkers for Patients with Intraductal Papillary Mucinous Neoplasms of the Pancreas. Cancer Prev. Res..

[17] Schultz N.A., Dehlendorff C., Jensen B.V., Bjerregaard J.K., Nielsen K.R., Bojesen S.E., Calatayud D., Nielsen S.E., Yilmaz M., Holländer N.H. (2014). MicroRNA biomarkers in whole blood for detection of pancreatic cancer. JAMA.

[18] Vila-Navarro E., Duran-Sanchon S., Vila-Casadesús M., Moreira L., Ginès À., Cuatrecasas M., Lozano J.J., Bujanda L., Castells A., Gironella M. (2019). Novel Circulating miRNA Signatures for Early Detection of Pancreatic Neoplasia. Clin. Transl. Gastroenterol..

[19] Matthaei H., Wylie D., Lloyd M.B., Molin M.D., Kemppainen J., Mayo S.C., Wolfgang C.L., Schulick R.D., Langfield L., Andruss B.F. (2012). miRNA biomarkers in cyst fluid augment the diagnosis and management of pancreatic cysts. Clin. Cancer Res..

[20] Lee L.S., Szafranska-Schwarzbach A.E., Wylie D., Doyle L.A., Bellizzi A.M., Kadiyala V., Suleiman S., Banks P.A., Andruss B.F., Conwell D.L. (2014). Investigating MicroRNA expression profiles in pancreatic cystic neoplasms. Clin. Transl. Gastroenterol..

[21] Wang J., Paris P.L., Chen J., Ngo V., Yao H., Frazier M.L., Killary A.M., Liu C.G., Liang H., Mathy C. (2015). Next generation sequencing of pancreatic cyst fluid microRNAs from low grade-benign and high grade-invasive lesions. Cancer Lett..

[22] Frampton A.E., Castellano L., Colombo T., Giovannetti E., Krell J., Jacob J., Pellegrino L., Roca-Alonso L., Funel N., Gall T.M.H. (2015). Integrated molecular analysis to investigate the role of microRNAs in pancreatic tumour growth and progression. Lancet.

[23] Desmond B.J., Dennett E.R., Danielson K.M. (2019). Circulating Extracellular Vesicle MicroRNA as Diagnostic Biomarkers in Early Colorectal Cancer-A Review. Cancers.

[24] Zhang L., Wang X., Chen P. (2013). MiR-204 down regulates SIRT1 and reverts SIRT1-induced epithelial-mesenchymal transition, anoikis resistance and invasion in gastric cancer cells. BMC Cancer.

[25] Qiu Y., Wei Y., Shen N., Wang Z., Kan T., Yu W., Yi B., Zhang Y. (2013). miR-204 inhibits epithelial to mesenchymal transition by targeting slug in intrahepatic cholangiocarcinoma cells. Cell Physiol. Biochem. Int. J. Exp. Cell Physiol. Biochem. Pharmacol..

[26] Lodygin D., Tarasov V., Epanchintsev A., Berking C., Knyazeva T., Körner H., Knyazev P., Diebold J., Hermeking H. (2008). Inactivation of miR-34a by aberrant CpG methylation in multiple types of cancer. Cell Cycle.

