# Supplementary material for: Micro-RNA Analysis of Pancreatic Cyst Fluid for Diagnosing Malignant Transformation of Intraductal Papillary Mucinous Neoplasm by Comparing Intraductal Papillary Mucinous Adenoma and Carcinoma
Source: J Clin Med. 2021 May 22;10(11):2249. doi: 10.3390/jcm10112249 (PMC8196884; doi:10.3390/jcm10112249)
Supplement: Supplementary file 1 [file jcm-10-02249-s001.zip › jcm-1165311-supplementary.pdf]

Table S1. Clinical and pathological features of IPMN lesions comparing IPMA with IPMC.

Items	IPMA	IPMC
Number of patients, n	8	4
Age, years, median (range)	73.5 (68-76)	67.5 (48-76)
Gender, male/female, n	5/3	2/2
IPMN lesions		
Site of lesions, head/neck/body/tail/MPD, n	2/1/4/1/0	0/0/2/1/1
Diameter of the lesions, mm, median (range)	38.5 (18-54)	40 (34-75)
Presence of septum, n	6	3
Presence of nodule, n	5	2
Communication with MPD, n	8	2
Serum CA19-9, U/ml, median (range)	6.4 (0.1-19.4)	131.65 (15-4500.7)

IPMA, intraductal papillary mucinous adenoma; IPMC, intraductal papillary mucinous carcinoma; IPMN, intraductal papillary mucinous neoplasm; MPD, main pancreatic duct
